# Toward ‘Vaccine Internationalism’: The Need for an Equitable and Coordinated Global Vaccination Approach to Effectively Combat COVID-19

**DOI:** 10.3389/ijph.2021.1604077

**Published:** 2021-04-14

**Authors:** Brian L. H. Wong, Manfred S. Green, John Reid, Jose M. Martin-Moreno, Nadav Davidovitch, Laurent Chambaud, Lore Leighton, Mohamud Sheek-Hussein, Ranjeet Dhonkal, Robert Otok, John D. Middleton

**Affiliations:** ^1^ MRC Unit for Lifelong Health and Ageing at UCL, University College London, London, United Kingdom; ^2^ Vaccination Group, COVID-19 Task Force, Association of Schools of Public Health in the European Region (ASPHER), Brussels, Belgium; ^3^ School of Public Health, Faculty of Social Welfare and Health Sciences, University of Haifa, Mount Carmel, Israel; ^4^ Department of Public Health and Wellbeing, University of Chester, Chester, United Kingdom; ^5^ Department of Preventive Medicine and INCLIVA, University of Valencia, Valencia, Spain; ^6^ Honours Committee, Association of Schools of Public Health in the European Region (ASPHER), Brussels, Belgium; ^7^ Department of Health Systems Management, Ben-Gurion University of the Negev, Beersheba, Israel; ^8^ École des Hautes Etudes en Santé Publique, Rennes, France; ^9^ Secretariat, Vaccination Group, COVID-19 Task Force, Association of Schools of Public Health in the European Region (ASPHER), Brussels, Belgium; ^10^ College of Medicine and Health Sciences, United Arab Emirates University, Al-Ain, United Arab Emirates; ^11^ Department of Health Sciences, Faculty of Life Sciences, Hamburg University of Applied Sciences, Hamburg, Germany; ^12^ Executive Board, Association of Schools of Public Health in the European Region (ASPHER), Brussels, Belgium

**Keywords:** COVID-19, vaccination, equity, global health, vaccine distribution, policy, global governance, vaccine policy

The prompt achievement of (vaccine-derived) herd immunity worldwide should be the core aim of all COVID-19 vaccination programmes, as it would thereby minimize viral transmission both between and within countries [1, 2]. Failure to address underlying structural and systemic inequalities in the acquisition and delivery of vaccinations is a fundamental ethical and moral concern. Additionally, there is a strategic imperative with pragmatic consequences in ensuring other vital goals of comprehensive global vaccination. These include: supporting sustainable economic development; allowing international travel/movement; and restoring adequate, accessible health and social care systems for all populations worldwide.

No region/nation will truly be free of the pandemic until all nations are free of it. Until an international consensus has been reached and a coordinated operational strategy has been adopted, the virus will find new vulnerable populations and continue to spread [3]. It will neither respect international boundaries, nor be limited/eradicated by sporadic vaccination coverage across populations and countries. It will continue to replicate and mutate to new, unpredictable forms, which potentially limit the effectiveness of current and future vaccines, and further threaten global health security and economic prosperity. Consequently, there will persist a need for restrictions on international travel, variably and inadequately implemented by individual countries.

ASPHER is deeply concerned about chaotic differences in vaccination policies between and within countries which threaten our collective ability to control and suppress the virus worldwide [4]. Given the potential for further virus mutations, some of which may be vaccine-resistant, the need for a coordinated global approach to vaccination through an equity lens has never been more evident. We need to overcome the ugly face of ‘vaccine nationalism’ and replace it with ‘vaccine internationalism’ [5] if we are to address the challenges impeding global access to COVID-19 vaccines (production, affordability, allocation, and deployment) [6].

ASPHER calls upon the WHO to lead efforts in securing global international collaboration and capacity (in immunology, virology, public health, and vaccinology) to anticipate potential antigenic drift/shift in the severe acute respiratory syndrome coronavirus 2 (SARS-COV-2) virus. It is crucial for the WHO to not only bolster its efforts to maintain and develop comprehensive vaccine responses to the virus, but also adapt them to combat potential new strains.

ASPHER calls upon national governments to commit to international vaccine leadership through the WHO, supported by other key international agencies and integrated with the COVAX GAVI initiative. National governments must adopt an international consensus with a clear public health strategy and measurable targets for reducing the virus spread. Such a strategy requires recognition of the need for appropriate vaccine deployment, in accordance with local health system contexts and is not merely about obtaining funding to support poorer countries with vaccine access [7]. ASPHER further calls on national governments to contribute to a coordinated international effort to ensure vaccine deployment programmes are comprehensive and fit for purpose, with considerations made regarding the circumstances within countries in which they are administered. Additionally, we call on national governments to strengthen issues around anticipating new variants and decreasing effectiveness of vaccines, proactively planning for if/when vaccines stop working to prevent mild/moderate disease as well as considering how countries are reimbursed for vaccines purchased which may be less effective. Finally, it is essential that national governments evaluate their need to access global vaccine stockpiles, bearing in mind the context of global equity.

Global surveillance and disease epidemiology, rapid reporting of vaccine delivery and uptake with planned seroprevalence studies, and support for alerting and mobilizing outbreak control for emergent diseases within the rapid reporting dashboard are crucial components of a coordinated global approach. Therefore, ASPHER calls upon the WHO and national public health agencies to develop an international nomenclature for current and future virus mutations as well as urgently revise and agree on global evaluation frameworks for COVID-19 vaccines, building upon historical and recent pandemic-related approaches to communicable disease control, elimination, and eradication. We further call for the reporting of funding levels backed with COVAX pledges from countries and timely delivery of vaccines as well as the facilitation of post-marketing surveillance, including that of pharmaceutical companies.

ASPHER also calls upon civil society organisations (CSOs) and schools of public health to support the WHO and national governments in catalyzing efforts to combat the spread of disinformation and misinformation. CSOs and schools of public health must not only continue to convey factual, evidence-based information, but also identify and share best practices and expertize with policymakers.

ASPHER strongly believes the creation of a range of vaccines to combat SARS-COV-2 is a major scientific achievement brought about through truly international efforts. We reinforce our stance on the need for transparent and evidence-based decision making in policy, particularly with respect to vaccination approaches [8]. The willingness of scientists to collaborate must now be matched by politicians from all nations. Without coordinated global capacity and collaboration to anticipate new virus strains and new vaccine modifications, we may be faced with a perpetual COVID pandemic. As such, the costly ‘vaccine nationalism’ we have seen thus far must not persist [9].

Politicians of all nations must commit to a global strategy for the control and eventual elimination of the SARS-COV-2 virus. There must be a genuine commitment to ensuring equitable access to vaccines both between and within countries. This is more than just a fundamental question of fairness and the right of all global citizens to health. The virus will not be eliminated anywhere, if it is not eliminated everywhere. Restoring the pursuit of health and better socioeconomic futures for all requires global commitment to the largest vaccination program to date. For this to succeed, nations will need to pool their resources and sovereignty, and put their weight behind the WHO’s leadership.

Recent disputes between the EU and the United Kingdom on vaccine distribution [10] drastically undermine effective vaccine rollout globally, doing more harm than good. Political leaders have set high expectations among their citizens to ensure rapid vaccine rollouts; however, limitations in technical production are inherent to all vaccine production efforts. While we have seen extraordinary success so far and so quickly, it is important to bear in mind that disinformation and misinformation will persist, and there are both commercial and political factors at play which can exacerbate existing health inequities [1]. Vaccine internationalism requires measured calm heads, diplomacy, and generous visionary leadership. Our aim above everything else as a global community needs to be defeating the virus first.
